# Enlargement of the Spleen—X

**Published:** 1908-10-17

**Authors:** 


### ENLARGEMENT OF THE SPLEEN—X.

### ENLARGEMENTS IN WHICH THE BLOOD CHANGES ARE NOT DISTINCTIVE.

(*Concluded.*)

*Splenic Anæmia and Banti's Disease.*—There is some doubt as to whether there is really any such disease as splenic anæmia; for in many of the cases that have been so diagnosed it has ultimately turned out that the liver has been cirrhotic. Cirrhosis of the liver, in which the earlier symptoms have been splenomegaly and simple anæmia, is Banti's disease.

True splenic anæmia, if it exists at all, is rare. It is described as a disease characterised by anæmia and seemingly idiopathic enlargement of the spleen. The latter may become very greatly enlarged, weighing from two to eight or ten pounds, but it seldom attains the absolutely huge size that it may do in spleno-medullary leuchæmia. The capsule is thickened and often adherent by peritoneal adhesions to adjacent structures; the trabeculæ are thickened, the Malpighian bodies small and fibroid, and the pulp diminished. It is said to affect young adult males chiefly; it is slowly progressive towards a fatal termination, which usually occurs within three years of the beginning of symptoms. There may be irregular periods of slight pyrexia, and occasionally hæmorrhages, especially from the stomach, and attacks of pain and tenderness in the left hypochondrium. It is most likely to be mistaken for spleno-medullary leuchæmia, cirrhotic liver, or ague-cake spleen. There is no leucocytosis, however, and this at once distinguishes it from leuchæmia, and the absence of glandular enlargement differentiates it from Hodgkin's disease. The anæmia is of the secondary type. The red cells become much reduced, though seldom to below 2,500,000 per cub. mm. The hæmoglobin is usually much more reduced, so that the colour index may be 0.5 or even less. Not only is there no leucocytosis—there is often leucopenia.

*Hodgkin's Disease, Lymphadenoma.*—There is a whole series of cases, differing from one another mainly in their relative acuteness or chronicity, but also in the extent to which the spleen is affected more than the lymphatic glands, or *vice versa;* and although there is some evidence to show that these cases all constitute members of the same main disease, there are different names given to certain chief types of the complaint. If there is general enlargement of the lymphatic glands throughout the body, little or no enlargement of the spleen, no special blood changes, and a rapidly fatal ending, the condition is termed *lymphosarcoma.* If the lymphatic glands, either in one locality only, or throughout the body, show great enlargement, without obvious affection of the spleen, no special blood changes, and a relatively benign course, the disease is styled *lymphadenoma.* The type of the complaint which is termed *Hodgkin's disease* is characterised by enlargement of lymphatic glands, progressive anæmia, and enlargement of the spleen and liver. The course is occasionally acute, but more often it is chronic; nearly always the course is slowly but surely towards death, though the end may be postponed for many months or even several years.

The spleen is, as a rule, only moderately enlarged —weighing from a half to two pounds—but in exceptional cases it may be greatly increased in size, its lower border reaching well below the level of the umbilicus. It may be either smooth and uniform, or if it contains large deposits of new-formed lymphoid tissue, slightly nodular or uneven. It is usually hard. On section, a typical Hodgkin or "hard-bake" spleen exhibits white nodules of lymphadenomatous growth standing out in sharp contrast to the normal dark red splenic pulp. The liver also is nearly always uniformly enlarged to a moderate extent, its surface being smooth and its edge sharp, well-defined and distinctly palpable below the costal margin. Groups of enlarged glands may be seen and felt, in the cervical and axillary regions most commonly, less often in the groins as well. There may be evidence of obstruction to veins or to a bronchus by enlarged mediastinal glands. At first the affected glands are soft, but later they become firm, and they give rise to large tumours and deformity. The skin, even if it is tightly stretched over these tumours, rarely becomes adherent to them; the glands do not become adherent to one another, nor fixed to the deeper structures; and it is very rarely that any of them suppurate—an important point of distinction between them and tuberculous glands.

The blood may show little or no departure from normal in the early stages of the disease, and at no time is the blood count pathognomonic. As time goes on the red corpuscles become gradually diminished to 3,000,000 or even 2,000,000 per cub. mm. The hæmoglobin is always more diminished than are the red cells, so that the colour index is always less than 1, and may be very low.

Leucocytosis only occurs when some intercurrent inflammation sets in — pleurisy, for example. The only service of the blood count, therefore, is to exclude lymphatic leuchæmia, which Hodgkin's disease may closely simulate, except that there is marked leucocytosis in the former complaint and little or none in the latter. The differential leucocyte count in an uncomplicated case of Hodgkin's disease is often normal, though an occasional basophile cell or myelocyte may be seen. Sometimes the small lymphocytes may be relatively increased to a slight extent; whilst with intercurrent inflammation the proportion of polymorphonuclear cell rises.

We would conclude with four aphorisms:—

1. Big spleen, big glands, great lymphocytosis = lymphatic leuchæmia.
2. Big spleen, big glands, no leucocytosis = Hodgkin's disease.
3. Huge spleen, no big glands, great leucocytosis with myelocytes = spleno-medullary leuchæmia.
4. Big spleen, no big glands, no leucocytosis = splenic anæmia; which often turns out to be an initial stage of cirrhosis of the liver = Banti's disease.

